# Primary hepatic carcinoid tumor: case report and literature review

**DOI:** 10.1590/S1679-45082014RC2745

**Published:** 2014

**Authors:** Éden Sartor Camargo, Marcelo de Melo Viveiros, Isaac José Felippe Corrêa, Laercio Robles, Marcelo Bruno Rezende

**Affiliations:** 1 Hospital Santa Marcelina, São Paulo, SP, Brazil.

**Keywords:** Liver neoplasms, Neuroendocrine tumors/diagnosis, Carcinoid tumor, Case reports

## Abstract

Primary hepatic carcinoid tumors are extremely rare neoplasms derived from hormone-producing neuroendocrine cells. It is difficult to make their diagnosis before biopsy, surgical resection or necropsy. A recent publication described only 94 cases of these tumors. There is no sex predilection and apparently it has no association with cirrhosis or preexisting hepatic disease. The most effective treatment is hepatectomy, and resection is determined by size and location of the lesions.

## INTRODUCTION

The liver is the organ most often affected by metastases of neuroendocrine tumors,^(1-3)^ but the primary hepatic carcinoid tumors are extremely rare. Only 60 cases had been described up to 2008^(4)^ and 94 cases were reported in a recent publication. Carcinoid tumors were first reported by Lubarsch, in 1888,^(5)^ and are functional neoplasms that can develop in any part of the body. However, approximately 54% to 90% of the cases originate from the gastrointestinal tract,^(6,7)^ mainly from the appendix, small bowel and rectum.

The primary hepatic carcinoid tumors are neoplasms derived from hormone-producing neuroendocrine cells, especially serotonin. Since they are very rare, it is difficult to make their diagnosis before biopsy, surgical resection or even necropsy.^(8)^ There is no sex predilection and the tumors affect individuals aged between 40 and 50 years.^(9)^ The cases reported present large tumors in the hepatic parenchyma, with non-specific and mild symptoms. Due to fast progression of the tumor and delay in making diagnosis, very few cases are surgically resectable.^(10)^

The primary carcinoid tumor of the liver was first described by Edmonsdson, in 1958, and its origin remains uncertain.^(11)^ The mutating cells originate from the ectopic pancreatic or adrenal tissue in the hepatic parenchyma, or from neuroendocrine cells located in the intrahepatic biliary epithelium.^(12)^ Moreover, it was proposed that the chronic inflammation of the bile canaliculi can cause intestinal metaplasia, which predisposes the development of neuroendocrine tumors.^(12)^ The primary hepatic carcinoid tumor has no association with cirrhosis or preexisting liver disease^(4) ^and accounts to only 0.3% of the cases of neuroendocrine tumors.^(7)^

## CASE REPORT

A 34-year-old male patient referred abdominal pain that was intermittent, progressive, related to compression on the right flank and irradiating to the dorsum and ipsilateral shoulder, for three months. He presented postprandial fullness, constipation and weight loss (3% of body weight).

**Figure 1:**
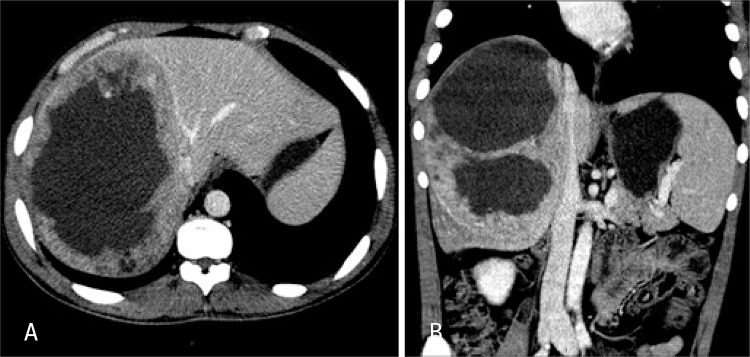
(A) Axial computed tomography image - portal phase - middle hepatic vein - contact with lesion. (B) Coronal computed tomography image

The laboratory test results showed normal complete blood count and liver function tests, negative serology for hepatites, slight increase in canalicular enzymes and tumor markers within normalcy. Abdominal ultrasound showed a heterogeneous mass in the right hepatic lobe measuring 12x10cm, with a solid-cystic content. Computed tomography demonstrated enlarged liver due to the right hepatic lobe that presented an extensive infiltrating lesion with poorly-defined contours, a liquefied center and the periphery was partially enhanced by contrast medium. There was no further involvement of other organs (Figure 1A and B).

**Figure 2:**
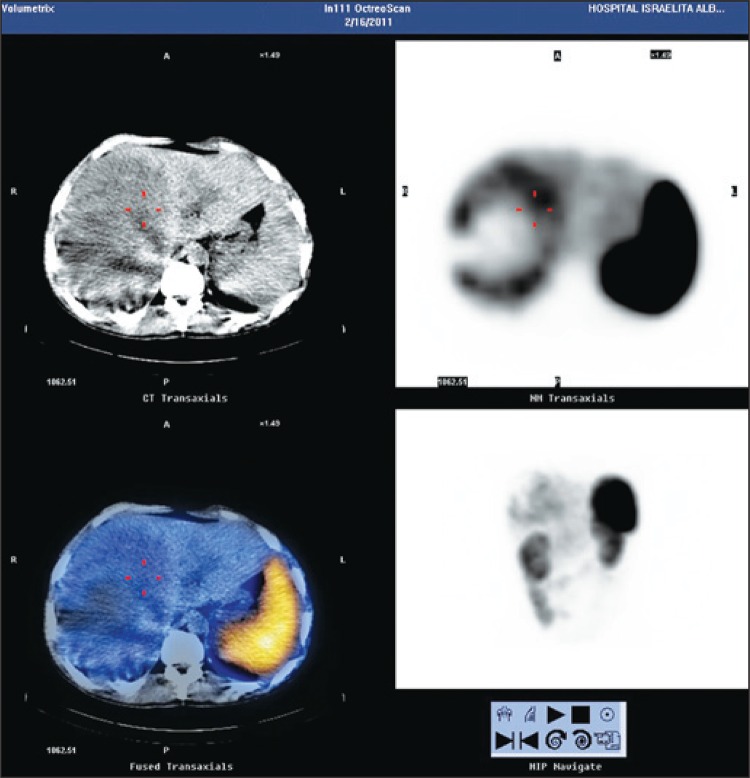
Octreotide scintigraphy shows radiomarker uptake in hepatic lesion

The patient was submitted to ultrasound-guided percutaneous liver biopsy. The pathologic report described neoplasm of undetermined histogenesis, suggesting carcinoid tumor, and immunohistochemistry confirmed the diagnosis. The investigation was complemented with OctreoScan^®^ that specifically demonstrated a primary hepatic carcinoid tumor (Figure 2).

**Figure 3:**
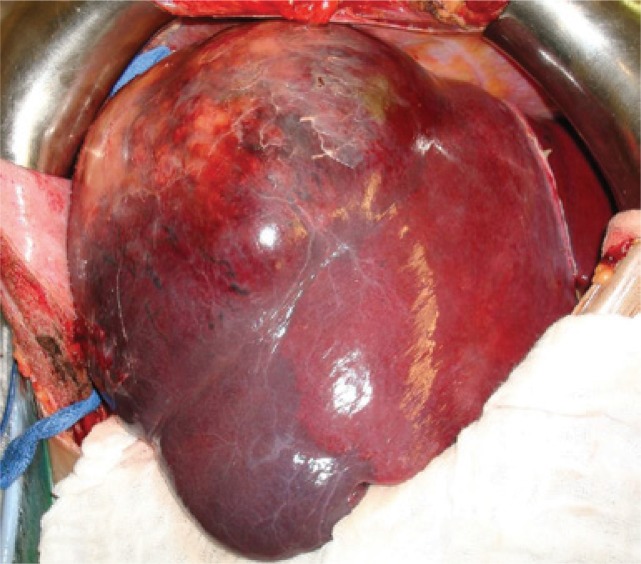
Liver with extensive lesion in the right lobe

**Figure 4:**
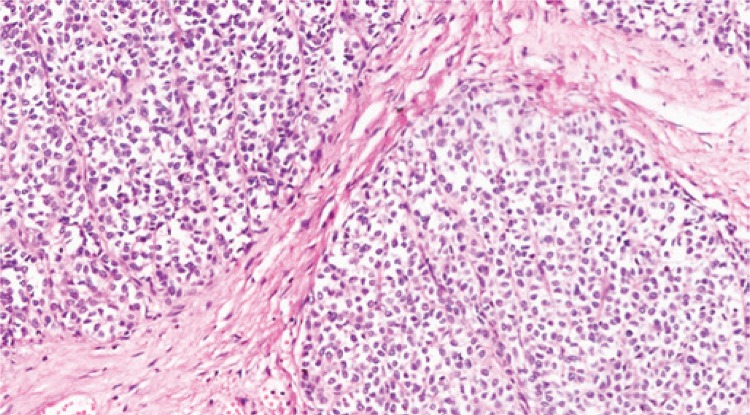
Histology – epithelioid neoplasm, with medium-sized cells, irregular nuclei, in organoid arrangements and blocks - 8 mitosis per 10 HPF, with necrotic foci

He underwent right hepatectomy, and an irregular extensive lesion occupying the whole right lobe was evident in the intraoperative period (Figure 3). The patient progressed well and was discharged on postoperative day 7. The pathological examination showed a malignant neoplasm with neuroendocrine differentiation and positive immunohistochemistry for synaptofisin, vimentin, chromogranin and KI-67, thus confirming the diagnosis of primary hepatic carcinoid tumor (Figure 4).

## DISCUSSION

Primary hepatic carcinoid tumor may be an incidental finding or it may present with abdominal pain, jaundice, a palpable mass in the upper right quadrant of the abdomen, weight loss, Cushing syndrome and carcinoid syndrome.^(4,13)^ There is usually liver metastasis when carcinoid syndrome occurs (facial redness, abdominal pain and diarrhea). This clinical picture is very rare, and there are only two cases described in the medical literature.^(8,14)^

The investigation should first include an abdominal ultrasound, which often demonstrates a solid-cystic tumor.^(2)^ Further workup with computed tomography generally corroborates the ultrasonographic findings. It is difficult to make a definite diagnosis primarily due to its similarity to hepatocellular carcinoma. Finally, OctreoScan^®^ is very useful and its specificity achieves up to 83%.^(15)^

Histopathology reveals a malignant neoplasm, with neuroendocrine differentiation and eight mitotic figures per high-power field, but it is not specific for diagnosis of primary hepatic carcinoid tumor, according to the literature.^(2)^ In the present study, immunohistochemistry correlated with the literature and showed positive chromogranin in 89.1%, and synaptofisin in 55% of the cases.^(8)^

The most effective treatment is hepatectomy.^(4)^ Survival rate may increase from 29% to 78%.^(5)^ Systemic chemotherapy or chemoembolization showed uncertain results.^(2)^ The indication of liver transplant remains a dilemma, and some researchers suggest this therapy for patients with multiple lesions or in cases of disease with impaired liver function.^(16)^ Another possible treatment is radiofrequency.^(4)^

Recent studies reported a five-year survival of 74-78% in patients submitted to hepatectomy, and the recurrence rate was approximately 18%.^(17,18)^ According to Schwartz et al.,^(4) ^the distant metastases are not described when there is no hepatic involvement.

Up to 2009, in the largest series published in the literature, Lin et al.^(8)^ reported 94 cases and described abdominal pain as the most frequent symptom (44% of cases), followed by abdominal mass (14.3%) and absence of symptoms (13.1%); unilobar location (76.6%) and single lesion (62,8%). Hepatectomy was performed in 86.8% of their patients, and overall mortality was 25.5%.

Huang et al.^(2)^ are the authors of the largest series from a single center, describing 11 cases of primary hepatic carcinoid tumor within a 13-year period of analysis.

## CONCLUSION

Carcinoid tumors with liver metastasis are relatively common. However, the primary involvement of this organ is very rare. Better diagnostic methods are required to make a more precise preoperative differentiation between primary hepatic carcinoid tumor and hepatocellular carcinoma.

Primary hepatic carcinoid tumor should be suspected in patients with no chronic liver disease, normal alpha-fetoprotein levels and solid-cystic lesion in imaging tests, associated with diarrhea and abdominal pain.
