# Supplementary material for: Primary hepatic carcinoid tumor: case report and literature review
Source: Einstein (Sao Paulo). 2014 Oct-Dec;12(4):505–8. doi: 10.1590/S1679-45082014RC2745 (PMC4879921; doi:10.1590/S1679-45082014RC2745)
Supplement: Translation

# Translation: Tumor neuroendócrino primário do fígado: descrição de caso e revisão da literatura

**Authors:** Éden Sartor Camargo, Marcelo de Melo Viveiros, Isaac José Felippe Corrêa, Laercio Robles, Marcelo Bruno Rezende

**Affiliations:** 1 Hospital Santa Marcelina, São Paulo, SP, Brasil.

**Keywords:** Neoplasias do fígado, Tumores neuroendócrinos/diagnóstico, Tumor carcinoide, Relatos de casos

## Abstract

Tumores carcinoides primários do fígado são neoplasias derivadas de células neuroendócrinas produtoras de hormônios, extremamente raras e de difícil diagnóstico antes da biópsia, ressecção cirúrgica ou mesmo da necrópsia. Em recente publicação, descreveram-se apenas 94 casos dessa afecção. É uma doença sem predileção por sexo e que, aparentemente, não tem associação com cirrose, nem com doença hepática preexistente. O tratamento que se demonstra mais eficaz é a hepatectomia, cuja extensão da ressecção é determinada pelo tamanho e pela localização das lesões.

## INTRODUÇÃO

Apesar de o fígado ser o principal órgão acometido por metástases dos tumores carcinoides,^(1-3)^ os carcinoides primários desse órgão são extremamente raros, com descrição de apenas 60 casos até o ano de 2008^(4)^ e de 94 casos em publicação mais recente. Inicialmente reportado por Lubarsch em 1888,^(5)^ os tumores carcinoides são neoplasias funcionais que podem se desenvolver em qualquer parte do corpo. Entretanto, cerca de 54 a 90% dos casos têm sua origem no trato gastrintestinal,^(6,7)^ principalmente no apêndice cecal, intestino delgado e reto.

Os tumores neuroendócrinos primários do fígado (TNPF) são neoplasias derivadas de células neuroendócrinas produtoras de hormônios, notadamente a serotonina, que, principalmente por sua raridade, os tornam de difícil diagnóstico antes da biópsia, ressecção cirúrgica ou mesmo da necropsia.^(8)^ Não apresentam predileção por sexo e acometem indivíduos na faixa etária entre 40 e 50 anos.^(9)^ Os casos relatados se apresentam como tumorações de grandes dimensões no parênquima hepático, com sintomas inespecíficos e frustos, tornando ainda mais raros os casos passíveis de ressecção cirúrgica, devido à rápida progressão tumoral e à demora no diagnóstico.^(10)^

O TNPF foi inicialmente descrito por Edmonsdson no ano de 1958 e sua origem ainda permanece incerta.^(11)^ As células mutantes originam-se de tecido ectópico pancreático ou adrenal encontrado no parênquima hepático ou, então, de células neuroendócrinas localizadas no epitélio biliar intra-hepático.^(12)^ Além disso, também é proposto que a inflamação crônica dos canalículos biliares pode acarretar metaplasia intestinal, a qual predispõe ao desenvolvimento do tumor neuroendócrino.^(12)^ O TNPF não apresenta associação com cirrose ou doença hepática preexistente^(4) ^e corresponde a apenas de 0,3% dos casos de tumores carcinoides.^(7)^

## RELATO DE CASO

Paciente masculino de 34 anos de idade, com relato de dor abdominal há 3 meses, intermitente, progressiva, de caráter compressivo, em flanco direito, com irradiação para o dorso e ombro ipsilateral. Apresentava plenitude pós-prandial, constipação intestinal e perda ponderal de 3% do peso corpóreo.

Possuía hemograma e exames de função hepática normais, sorologias negativas para hepatites, aumento discreto das enzimas canaliculares e marcadores tumorais dentro da normalidade. A ultrassonografia abdominal evidenciava uma massa heterogênea em lobo hepático direito de 12x10cm, com componente sólido-cístico, e a tomografia computadorizada, fígado com dimensões aumentadas às custas do lobo hepático direito, apresentando extensa lesão infiltrativa com contornos mal definidos, com centro liquefeito e periferia parcialmente realçada ao meio de contraste, além de ausência de comprometimentos de outros órgãos (Figura 1 A e B).

**Figura 1:** (A) Tomografia computadorizada - corte axial - fase portal - veia hepática media - contato com a lesão. (B) Tomografia computadorizada - corte coronal

O paciente foi submetido à biópsia hepática percutânea guiada por ultrassonografia. O laudo histopatológico demonstrou tratar-se de neoplasia de histogênese indeterminada, sugerindo tumor carcinoide, e a imuno-histoquímica comprovou o diagnóstico sugerido. Investigação complementada com OctreoScan^®^ demostrou tratar-se especificamente de um TNPF (Figura 2).

**Figura 2:** Cintilografia com ocreotide - evidenciando captação de radiofarmaco em lesão hepática

Realizada hepatectomia direita regrada, com evidência, no intraoperatório, de extensa lesão irregular que ocupava todo lobo hepático direito (Figura 3). O paciente apresentou boa evolução e alta hospitalar no 7º dia. A anatomopatologia demonstrou se tratar de neoplasia maligna com diferenciação neuroendócrina e imuno-histoquímica positiva para sinaptofisina, vimentina, cromogranina e KI-67, confirmando o diagnóstico de TNPF (Figura 4).

**Figura 3:** Fígado com lesão extensa acometendo lobo hepático direito

**Figura 4:** Histologia - neoplasia epitelióide com células de tamanho intermediário, nucleos irregulares, dispostas em arranjos organóides e blocos - 8 mitoses por 10 cga com focos de necrose

## DISCUSSÃO

O TNPF pode ser um achado incidental ou, então, apresentar-se com sintomas de dor abdominal, icterícia, massa palpável em quadrante superior direito do abdome, perda ponderal, síndrome de Cushing e síndrome carcinoide.^(4,13)^ Quando ocorre a síndrome carcinoide (rubor facial, dor abdominal e diarreia), geralmente há metástase hepática. Essa apresentação clínica é bastante rara, com descrição de apenas dois casos na literatura.^(8,14)^

A investigação deve se iniciar com ultrassonografia abdominal, que demonstra, na maioria das vezes, um tumor sólido-cístico.^(2)^ A propedêutica adicional com tomografia computadorizada de abdome geralmente corrobora os achados ultrassonográficos. Além disso, ressalta-se a dificuldade em se estabelecer o diagnóstico definitivo devido, principalmente, à sua similaridade com o carcinoma hepatocelular. Finalmente, o auxílio do OctreoScan^®^ é bastante benéfico, apresentando especificidade que chega a 83%.^(15)^

A histopatologia revelava se tratar de neoplasia maligna, com diferenciação neuroendócrina, com oito figuras de mitose por campo de grande aumento e, assim como dados da literatura, não é específica para o diagnóstico de TNPF.^(2)^ A imuno-histoquímica, no presente estudo, demonstrou correlação com a literatura, evidenciando a positividade da cromogranina em 89,1% e sinaptofisina em 55% dos casos.^(8)^

O tratamento mais eficaz é a hepatectomia.^(4)^ Há possibilidade de aumento de sobrevida de 29% para até 78%.^(5)^ Quimioterapia sistêmica ou quimioembolização mostraram resultados incertos.^(2)^ A indicação do transplante hepático ainda é um dilema, sendo que algumas pesquisas sugerem essa terapia em pacientes com múltiplas lesões ou em casos de doença hepática com função prejudicada.^(16)^ Outra possibilidade terapêutica é a utilização de radiofrequência.^(4)^

Recentes trabalhos demonstraram sobrevida de 5 anos em 74 a 78% dos casos de pacientes submetidos a hepatectomia, com uma taxa de recidiva em torno de 18%.^(17,18)^ As metástases à distância, de acordo com Schwartz et al.,^(4) ^não são descritas sem envolvimento hepático.

Até o ano de 2009, Lin et al.,^(8)^ na maior casuística da literatura, reportaram 94 casos e evidenciaram dor abdominal como o sintoma mais frequente (presente em 44% dos casos), seguido por massa abdominal (14,3%) e ausência de sintomas (13,1%); localização unilobar (76,6%) e lesão única (62,8%). A hepatectomia foi realizada 86,8% de seus pacientes, com uma taxa de mortalidade global de 25,5%.

Huang et al.^(2)^ são os autores com a maior casuística de um único serviço, com relato de 11 casos de TNPF em 13 anos de análise.

## CONCLUSÃO

Os tumores carcinoides metastáticos para o fígado são relativamente comuns. Entretanto, o acometimento primário desse órgão é bastante raro. Necessita-se de melhora nos métodos diagnósticos para que haja uma diferenciação pré-operatória mais precisa entre tumores neuroendócrinos primários do fígado e carcinoma hepatocelular.

Pacientes sem um passado de hepatopatia crônica, com níveis normais de alfafetoproteína e imagem sólido-cística nos exames de imagem, combinadas com diarreia e dor abdominal, devem ter aventada a hipótese de um caso de tumor neuroendócrino primário do fígado.
